# Imaging findings of vinyl dimethyl polydimethylsiloxane used as a paraurethral injectable for female stress urinary incontinence

**DOI:** 10.1177/17562872211060909

**Published:** 2021-12-05

**Authors:** Allert M. de Vries, Fenne M. Casteleijn, Jan-Paul W.R. Roovers, John P.F.A. Heesakkers, Jurgen J. Fütterer

**Affiliations:** Department of Urology – Experimental Urology (Route 267), Radboud University Medical Centre, Geert Grooteplein 10, P.O. Box 9101, 6500 HB Nijmegen, The Netherlands; Department of Obstetrics and Gynaecology, Amsterdam UMC, Amsterdam, The Netherlands; Department of Obstetrics and Gynaecology, Amsterdam UMC, Amsterdam, The Netherlands; Department of Urology, Radboud University Medical Centre, Nijmegen, The Netherlands; Department of Radiology and Nuclear Medicine, Radboud University Medical Centre, Nijmegen, The Netherlands

**Keywords:** computed tomography, stress urinary incontinence, urethral bulking agent, urolastic

## Abstract

**Objectives:**

Vinyl dimethyl polydimethylsiloxane (VDPDMS) is a urethral bulking agent used for female stress urinary incontinence (SUI), that is clearly visible on computed tomography (CT). Clinical effects are promising, but it remains difficult to identify factors predicting clinical success. Clinical outcome might depend on the shape and position of the implants after injection. Objective of this study is to analyze the appearance and position of bulk material on CT scans and to see whether it is delivered the intended circumferential and mid-urethral position.

**Methods:**

A single-center retrospective study was performed in 20 women, treated with VDPDMS for SUI. A senior radiologist analyzed all CTs, using an assessment scheme. This scheme describes whether the bulk is scattered, mid-urethral, and/or circumferentially distributed. The imaging findings were subsequently correlated to the patient global impression of improvement (PGI-I) and the percentage of subjective improvement experienced 6 weeks post-operatively.

**Results:**

The patient’s mean age was 61 years, and they underwent median 2.0 previous surgical treatments for SUI. Three patients reported no improvement, 9 patients had 20–90% improvement and 8 reported >90% improvement of their SUI. In 17/74 (24%) positions, the implant was scattered rather than spherical. In 9/20 (45%), the implants were not located in the intended mid-urethral position. In 8/20 patients (40%), the material was distributed circumferentially.

**Conclusion:**

This is the first study describing the position and shape of VDPDMS in patients after treatment. The appearance and position of the implants appears to be variable, but optimal positioning or shape seems to be no absolute requisite for success.

## Introduction

Urethral bulking agents (UBAs) have been used for many years in the treatment of female stress urinary incontinence (SUI). Although the mid-urethral sling (MUS) remains the gold standard in the second-line treatment of SUI, there are still many cases where patients would be better treated in a less-invasive way. The less-invasive nature of bulking agents has to be weighed against a generally reported limited efficacy and need for re-injections because the efficacy diminishes over time.^1,2^ Selecting the right patients can be challenging, especially because the exact working mechanism of UBAs is unclear. Part of the effect of UBAs is explained by improved urethral coaptation,^3^ which leads to an increase in urethral resistance and therefore better continence. Mid-urethral support is important for reflex closure of the urethra.^4^ Another factor may therefore be (mid-)urethral support bulk provides, reducing hypermobility of the urethra often seen in women with SUI and improving sphincter function.

Since 2011, vinyl dimethyl polydimethylsiloxane (VDPDMS or Urolastic^®^), has been used for treatment of female SUI. This bulking agent differs from other bulking agents, because solid, non-resorbable and non-deformable implants are formed after injection. Most other bulking agents currently used consist of biodegradable gels or deformable substances. In theory, biodegradation and/or deforming of the implants may contribute to recurrence of incontinence over time. Because VDPDMS solidifies after injection, the risk of recurrence of incontinence could be lower.

In the first cohorts of patients treated with VDPDMS, 2-year improvement rates of 33–66% were achieved and dry rates of 22–45%, mainly in recurrent patients.^5,6^ In our own experience, VDPDMS treatment in certain patients was very effective, but other patients showed limited or no effect with this treatment.^7^ Since a blind injection technique is used and because erosion of bulking material through the vaginal wall was observed in several patients, the question arose whether VDPDMS always reaches the intended place. Clinical effectiveness might depend on the anatomical position, shape, and volume of the implants.

Current clinical practice is ideally, that 0.8–1.0 cc of material is injected circumferentially around the mid-urethra, adjacent to the urethral wall. After several minutes, solid spherical implants are formed. This causes co-aptation of the urethra, which should in turn enhance urethral support and resistance. Only scarce data about the position of bulk material related to clinical outcome is available.^8,9^ This data show, through the use of imaging, that circumferential distribution of bulking material around the urethra was more prevalent in women with successful treatment. It remains however hard to compare different bulking agents and imaging techniques.

Due to its radiopacity, VDPDMS is clearly visible on CT. Furthermore, this imaging technique is inexpensive and provides a 3D-image of the implants. The preferred imaging technique is low-dose CT, as it is carries a relatively low burden of ionizing radiation. Imaging with transvaginal or transurethral ultrasound is difficult because the material is very echogenic, as the acoustic shadow makes it impossible to investigate the exact position of the injectable.

For this study, we describe the appearance and position of VDPDMS on CT, to assess whether the blind injection procedure delivers the bulk in the intended position. These CT findings are subsequently correlated to the clinical outcomes to identify factors possibly predictive for success or failure.

## Materials and methods

We retrospectively analyzed the charts of all women who had received VDPDMS injections in the Radboud University Medical Center, a tertiary referral center in Nijmegen, The Netherlands. Patients with predominant SUI based on positive cough–stress test and/or urodynamic investigation were treated. Decision for VDPDMS was based on failure of previous treatments or patient preference. The procedures were performed between November 2013 and April 2017.

All patients who underwent CT imaging on which VDPDMS was visible were included in the study. The CTs were performed in every treated patient from 2015 onwards to evaluate the position of the implants between 1 and 6 weeks after the procedure. Patients with CTs that were not assessable were excluded, as were patients who received injections for other reasons than SUI, such as a leaking urostomy.

Patient data (age, medical history, pad-usage, and CT images) and procedure data (date, injected volume, complications, and clinical effect) were extracted from the electronical medical records (EMR) and anonymized by one of the authors, who was part of the treatment team. All patients in our tertiary referral center were asked in a written statement if they had any objection to the usage of their anonymised medical data for scientific research purposes. None of the patients made objection. A waiver from the ethical committee of the Radboud University Medical Center was obtained for usage of the anonymised data (2017-3424). The ethical committee was aware of the method of anonymization and approved the procedure.

Our primary outcome was the presence of bulk material at the intended position on CT imaging. Secondary outcomes were patient-reported subjective improvements of continence and complications. The subjective improvement was measured using the percentage of subjective improvement reported by all patients and the validated patient global impression of improvement (PGI-I), which was documented from 2015 onwards. Both outcomes were available at different follow-up times because of a simultaneously performed post-marketing study. The charts were reviewed to find complications, such as temporary retention, post-operative pain, erosion, and the need for removal of one of the implants.

### The procedure

**Figure 1.**
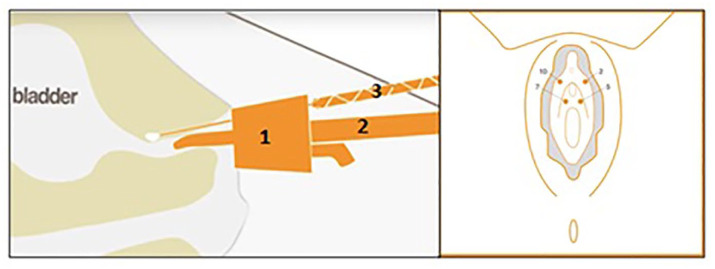
Urolastic-procedure and injection positions. Paraurethral injection of VDPDMS at 2, 5, 7, and 10 o’clock position at the level of the mid-urethra using applicator (1) and applicator support (2). The two components are combined in the static mixer (3).

Treatment with VDPDMS consists of an outpatient procedure in which four blind injections are given under local anesthesia using the Urolastic-applicator and the applicator support (Figure 1). No cystoscope is used. The applicator is fixed on the support based on the length of the urethra. The applicator facilitates the delivery of the material in the intended position that is, paraurethrally at the level of the mid-urethra at the 2, 5, 7, and 10 o’clock positions (Figure 1). In uncomplicated cases, four times 0.8 mL was injected. Depending on patient requirements and anatomy, the volume per position could be adjusted to 0.6–1.2 mL.

**Figure 2.**
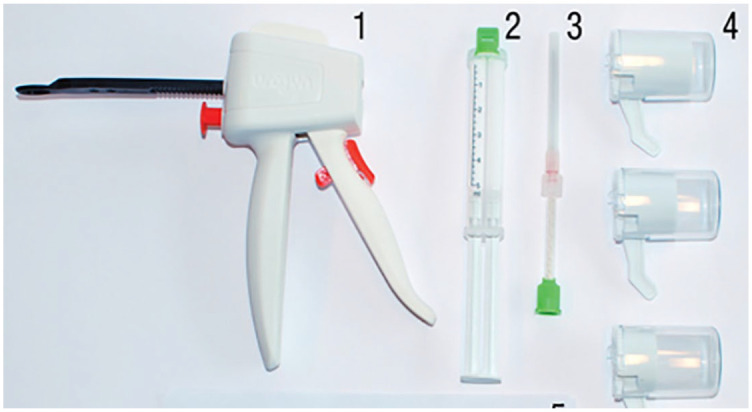
Urolastic hardware. Application gun (1) syringe with two components (2) static mixer with needle (3) different sizes of applicators (4).

During the injection with a gun, two components are combined in a static mixer (Figure 2). After mixing, the material is injected through a needle and hardens within 3–4 minutes into a solid, rubberlike implant. The shape of the implant is intended to be spherical to reduce the risk of pain from ‘sharp’ extensions.

Finally, a cough–stress test is performed to confirm a satisfactory effect on SUI. All procedures were performed by two right-handed urologists, trained in this procedure.

### CT assessment

A senior radiologist with experience in the field of urogynaecology assessed all CTs using a systematic scheme (Appendix 1) that was partially based on the method used by Hegde *et al.*^9^ for Macroplastique^®^. Although Macroplastique has different properties than Urolastic, this systematic scheme, using the division of the urethra into quadrants and into proximal, distal and mid-urethral sections, was useful in the assessment of the CT scans. The assessment was extended to describe the shape of the implants. This is relevant since, in the case of surgical removal, a non-spherical implant was found on multiple occasions. In these cases, the bulk material seemed scattered or was following anatomical structures such as small blood vessels. The scheme thus aimed at verifying whether VDPDMS is present as a solid spherical implant, circumferentially, at the level of the mid-urethra.

**Figure 3.**
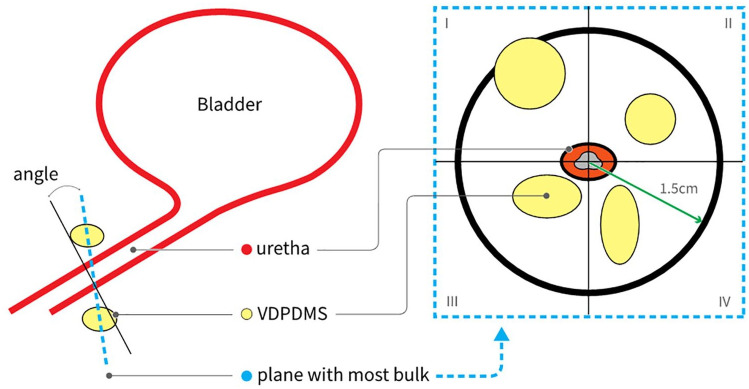
Defining the plane with most VDPDMS present. Schematic sagittal view of the bladder and urethra (left) and a cross-section (right) in the plane with most VDPDMS present, divided into two quadrants.

Initially, possible scattering of the material (i.e. a non-spherical implant), injection in a lymph or blood vessel and presence of VDPDMS >1.5 cm from the urethra were observed; thereafter, the plane and the angle of the plane in which most material was present around the urethra were defined (as depicted in Figure 3). The angle of the plane represents the number of degrees of deviation from the plane perpendicular to the urethra.

Subsequently, the urethral length was measured. If the plane with the most bulk was located in the middle third of the urethra, it was considered to be ‘mid-urethral’. The distance of the most proximal part of the implants from the urethrovesical junction was then measured. To assess whether the VDPDMS was distributed equally around the urethra, the surface of the plane occupied by VDPDMS within a radius of 1.5 cm from the center of the urethra was measured in four quadrants (Figure 3). If VDPDMS was present in all four quadrants, the material was considered to be circumferentially distributed. Finally, the volume of the material was measured to make a comparison with the documented amount of injected VDPDMS. Sometimes during the procedure, material is lost because of backflow through the tract of the injection needle, so it seemed relevant to make this comparison.

The calculations on the images were made with commercially available software from TeraRecon (Foster City, California, USA).

### Statistical analysis

The study was not powered for obtaining statistically significant correlations, but these were calculated to identify potential predictors for subjective improvement or complications. Correlations were calculated between the CT parameters and all clinical outcomes, using Spearman’s Rho. *P* values were determined to assess statistical significance, with a significance level (α) of 5%. Multiple regression was not feasible considering the small study population. A linear regression with backward reduction was performed to identify CT findings that are possible predictors for clinical success or complications.

All statistical analyses were made with Excel for Windows and IBM SPSS statistics for Windows, version 22.0 from IBM Corp. (Armonk, New York, USA).

## Results

### Patients

Twenty females were included, with CT imaging after injections with VDPDMS. One patient was excluded because the quality of the scan was too low for the required analysis. The patient characteristics are depicted in Table 1. The mean age was 61.1 years (SD ± 8.5) at the time of the procedure. Previous treatments were defined as any surgical treatment influencing continence and/or prior treatments for urge-incontinence or pelvic organ prolapse. The median number of previous treatments these women had undergone prior to injection was 2.0 (interquartile range (IQR) 4.0), reflecting the large number of recurrent patients in our tertiary referral center. The median number of pads used per 24 hours was 3.8 at baseline (IQR 2.8).

**Table 1.** Patient characteristics.

Characteristic	(*N* = 20)
Age in years (mean)	61.1 (±8.5; 43–77)^a^
Previous surgical SUI treatments (median)	2.0 (4.0; 0–9)^b^
Mid-urethral slings	21
Bulking agents (Macroplastique, Bulkamid^®^)	11
Pad usage before treatment (median)	3.8 (2.8; 1–6.5)^b^
Pad usage after treatment (median)	1.0 (3.0; 0–5)^b^
Injected volume in milliliters (mean)	3.1 (±0.8; 1.2–4.4)^a^
Subjective improvement % (median)	80.0% (68.8; 0–100%)^b^
Subjective improvement PGI-I:	
Very much better	7
Much better	3
Little better	2
No change or worse	1
Complications:	
Pain	9
● Left-sided pain	2
● Right-sided pain	3
Erosion	3
Temporary retention	5
Removal of material	7

PGI-I, patient global impression of improvement; SUI, stress urinary incontinence.

a (±Standard deviation (SD); range).

b (Interquartile range (IQR); range).

### Clinical effect

In 17 out of 20 women, the VDPDMS was injected in four positions, with a median of 0.8 mL per position. Two women received injections only ventrally of the urethra (i.e. at 5 and/or 7 o’clock) because other previous procedures made dorsal injections not feasible. Although these two patients had a negative cough–stress test after the procedure, the continence on follow-up was poor. One woman was continent during a cough–stress test performed after three injections, after which the procedure was terminated. This women experienced 80% improvement.

The median subjective improvement at 6 weeks follow-up was 80.0%. Eight patients reported a subjective improvement of >90%, and nine patients reported a subjective improvement of their incontinence of 20–90%. The remaining three experienced no improvement, or experienced worsening of their incontinence. Among this group with no improvement were the two women who received injections only ventrally.

The PGI-I showed an improvement in 12 out of 13 patients (92%). Pad usage was available for 12 women pre-operatively and 15 women post-operatively. The median pad usage per 24 hours decreased with 2.8–1.0 pad.

Complications of some sort were seen in 14 of 20 patients. Most complications were mild and were resolved within days. Post-operative pain was reported by 9 of 20 patients. In seven patients, post-operative pain or discomfort led to removal of bulking material. Removal consisted of a small vaginal incision under local or regional anesthesia, after which the VDPDMS can be removed with a forceps. Five of 20 patients experienced temporary urinary retention for which an indwelling catheter was placed for 24 hours. There were no cases of permanent urinary retention observed.

### CT findings

#### Scattering

The first part of CT assessment consisted of determining the shape of the implants per injection position. In Table 2, the positions at which scattering occurred are summarized. Scattering occurred in 17 of 74 injections (23%) and mostly at the 10 and 2 o’clock position (total 11 times in these positions). In 11 out of 20 patients (55%), the material was scattered in one or more positions. No correlation was found between scattering and subjective improvement or complications.

**Table 2.** CT findings.

	Scattering			Following anatomy		
	Scattered implants (no)	Total implants (no)	Percentage scattered	Implants following anatomy (no)	Total implants (no)	Percentage following anatomy
10 o’clock	7	17	41	8	17	47
2 o’clock	4	18	22	13	18	72
5 o’clock	3	19	16	7	19	37
7 o’clock	3	20	15	3	20	15
Total	17	74	23	31	74	42

CT, computed tomography.

#### Following anatomy

In almost all patients (19/20), the injected material seemed to follow the structure of small blood or lymph vessels or endopelvic fascia in at least one of the four positions. Examples can be found in Appendix 2. In Table 2, the positions at which implants were observed are specified. The 2 o’clock position was most at risk for this finding, 13 out of 18 implants (72%) were not entirely spherical. The close relation of VDPDMS to anatomical structures was not associated with complications such as pain or erosion and did not influence subjective improvement of urinary incontinence.

### Position relative to the urethra

The distance between VDPDMS and the middle of the urethral lumen was measured. In 7 of 74 injection sites (9%), the VDPDMS was situated more than 1.5 cm from the urethral lumen. No correlation was found between pain or other complications and the distance to the lumen.

In Table 3, the findings of the assessment of other variables investigated are summarized. Since the urethra of one patient was not entirely scanned, no exact urethral length could be given. It was however possible to conclude that VDPDMS was located mid-urethrally.

**Table 3.** CT characteristics and clinical outcome after VDPDMS.

Patient	Angle of plane with most bulk	Urethra length (mm)	Mid-urethral (yes/no)	Distance bladder-neck (mm)	Circumferential (yes/no)	Subjective improvement (%)
1	12	32	Y	12	N	0
2	13	32	Y	9	Y	25
3	18	32	Y	9	Y	20
4	12	32	N	0	N	0
5	18	34	N	0	Y	100
6	11	34	Y	6	N	100
7	15	33	N	4	N	100
8	6	31	N	3	N	50
9	15	29	N	0	Y	0
10	12	39	Y	18	Y	70
11	11	30	Y	11	N	99
12	55	34	N	21	N	80
13	14	34	Y	13	N	100
14	22	38	Y	13	N	80
15	14	36	N	20	N	70
16	–	–	Y	14	Y	90
17	0	40	Y	16	N	99
18	4	44	N	13	Y	100
19	10	40	N	3	N	100
20	12	44	Y	18	Y	80

CT, computed tomography; VDPDMS, vinyl dimethyl polydimethylsiloxane.

The angle of the plane in which the most bulk was visible was significantly larger with increasing age. This correlation was moderate (ρ.65; *p* value .002). The urethral length as measured from the CT scan, was positively correlated with the percentage of subjective success, (ρ.53; *p* value .02).

In 11 out of 20 patients (55%), the bulk was located mid-urethrally. No significant correlation was found between a mid-urethral position (ρ-.05; *p* value .82) and clinical success. The distance of the most proximal implant to the bladder neck was 0 mm in 3 out of 20 patients (15%). In another four patients, this distance was short: 3–6 mm. The positioning of VDPDMS at the urethrovesical junction was not correlated with clinical success (ρ.05; *p* value .82) or complications such as pain or erosion.

In 8 of the 20 patients (40%), the material was distributed circumferentially; that is, present in all four quadrants. This type of distribution did not correlate with subjective success(ρ-.04; *p* value .87).

The images in Appendix 2 show the variability in distribution of VDPDMS. Less than half of the material could be traced with volume measurement. The mean documented total volume of injected VDPDMS was 3.1 mL. The CT measurements resulted in 1.3 mL.

### Predictors

To identify possible predictors for clinical success, a linear regression analysis with backward reduction was performed. A longer urethral length was the only variable able to predict success, at 51.4%. All other investigated parameters mentioned in Table 3 had no significant predictive value.

## Discussion

The assessment scheme used, enabled us to systematically evaluate the appearance of VDPDMS on CT. The position and distribution of VDPDMS was highly variable. In 45% of the patients, the material was not positioned at the mid-urethra. In 60% of the patients, the distribution was not circumferential. A non-intentional position and distribution was not associated with a smaller subjective improvement. Of the other parameters investigated, only urethral length could be an indicator for clinical success based on the observations in this small group of patients. None of the parameters was significantly associated with complications.

Part of the explanation for the highly variable position and distribution of the bulking agent after injection is the delivery method. The procedure is not guided with imaging or cystoscopy. The physician therefore gets little feedback during the procedure about the position of the needle and the volume injected. Furthermore, injection is influenced by the resistance of the paraurethral tissue, which varies due to factors such as previous procedures or hormonal status. Injected material can sometimes flow back through the injection canal during the procedure, leading to an overestimation of the volume injected.

The discrepancy between injection volumes found on CT scans and the documented volumes could not be confirmed by our analysis. As described earlier, only half of the material could be measured in the CT scans with the used software. Also, it is difficult to measure the exact amount of fluid injected due to the shape of the implants after solidifying. For VDPDMS, volume measurement is however less relevant as it does not shrink and is not biodegradable.

We found a subjective improvement of 69% in the patient group with a mid-urethral positioning of VDPDMS, compared to 67% in the other patients. Unintended presence of the injectable at the urethrovesical junction did not seem to influence the outcomes negatively. Kuhn *et al.*^10^ performed a prospective study with periurethral collagen injections and concluded that mid-urethral injection is slightly favorable compared to bladder neck placement. In this study, 30 elderly women were divided into two groups receiving either a mid-urethral or bladder neck injection. No imaging was performed to confirm the anatomical placement of the collagen. The continence rates significantly differed between both groups with 66.6% and 60%, respectively. In analogue and considering the success of the MUSs, positioning of injectables at the mid-urethra seems logical. However, an ultrasound study in 100 patients treated with Macroplastique by Hegde *et al.*^9^ concluded differently. Proximally located Macroplastique was associated with better clinical outcomes, especially in combination with circumferential periurethral distribution of the bulk. In 33% of the patients with no subjective improvement of incontinence after treatment, the implants were distributed circumferentially, according to our definition. In the group with maximal improvement, this percentage was 25%. It was therefore not surprising that we did not find a significant correlation between distribution and subjective success. In 2003, Defreitas *et al.*^8^ published a study of 3D ultrasound evaluation of 46 women treated with periurethral collagen injections. The patients were divided into satisfied (21 patients) and non-satisfied groups (25 patients). One of the conclusions of this study was that circumferential distribution of the injectable correlated with the chance of a satisfactory continence status after the procedure. This correlation was however not absolute, as continence also occurred in women having an asymmetrical distribution of bulk. It is most likely that the same holds true for VDPDMS, but our study is too small to detect this. It would however correspond well with the clinical observation that one can still be continent after losing an implant. The urethra apparently does not need circular compression to improve the continence mechanism. Maybe the working mechanism of asymmetrical bulk is comparable to the MUS, enabling reflex closure of the urethra rather than causing coaptation.

Our study is limited by a low number of patients (20), which makes it difficult to find significant correlations between the parameters investigated and the clinical outcome. Furthermore the study is not powered to find significant correlations. Despite this, we identified several parameters that can be relevant for future research. One of the potentially clinically useful findings was the possible influence of length of the urethra. If this finding can be confirmed, it might lead to reconsideration of the therapeutical options in patients with a short urethra. The finding that the angle of the VDPDMS plane seemed larger in older women is also interesting. This might be the result of descensus of the pelvic organs including the proximal urethra and stresses the importance of determining the angulation of the urethra before injecting VDPDMS. It will be interesting to see the influence of these and other parameters in larger studies, designed to detect clinically significant parameters.

## Conclusions

CT provides a good way to visualize VDPDMS in vivo. The assessment scheme helped to systematically describe the appearance and position of the injectable. In case of VDPDMS, this appearance is highly variable. Non-intentional anatomical positioning of VDPDMS does however not always lead to clinical failure. In our study, urethral length seems relevant for predicting success, but the statistics of this study cannot confirm this finding. When we combine our series with other published results, it appears that the real correlation of positioning of bulk and clinical effect is still enigmatic.

## Figures and Tables

**Appendix 2.**
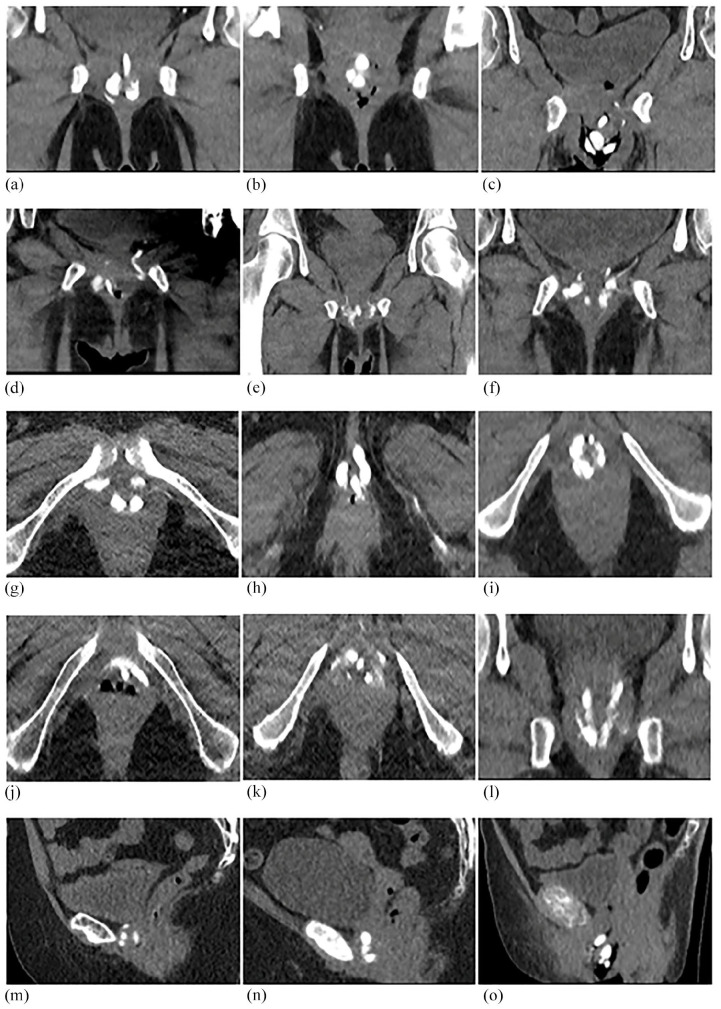
Appearance of VDPDMS on CT after treatment. (a–c) Coronal images, (d) coronal image with VDPDMS following anatomy, (e, f) coronal images with spreading, (g–i) axial images, (j, k) axial images with spreading, (l) coronal image with spreading, and (m–o) sagittal view.

## Appendix 1

### Score form

**Table:** Imaging Urolastic score form.

Study number: xxx Name observer: xxx Date observation: xx-xx-xxxx	
1. Observations:	
● Material scattered? at which position(s)?	Yes/No; at 2/5/7/10 o’clock
● Injection following anatomy? Position?	Yes/No; at 2/5/7/10 o’clock
● Material > 1.5 cm from urethra? Position?	Yes/No; at 2/5/7/10 o’clock
2. Angle of plane in which most Urolastic is deposited:	… degrees
3. Length of urethra	… mm
4. Plane in middle third urethra?	Yes/No
● Distance to bladder neck	… mm
5. Measuring amount of bulk by quadrant:	
● Area occupied by bulk quadrant I	… mm^2^/177 mm^2^;
● Area occupied by bulk quadrant II	… mm^2^/177 mm^2^;
● Area occupied by bulk quadrant III	… mm^2^/177 mm^2^;
● Area occupied by bulk quadrant IV	… mm^2^/177 mm^2^;
6. Circumferential with 4x > 1mm^2^bulk?	Yes/No
7. Volume of depositions:	
● 2 o’clock	… mm^3^
● 5 o’clock	… mm^3^
● 7 o’clock	… mm^3^
● 10 o’clock	… mm^3^
8. Remarks:	
